# Endothelial Function in Pulmonary Arterial Hypertension: From Bench to Bedside

**DOI:** 10.3390/jcm13082444

**Published:** 2024-04-22

**Authors:** Michele Correale, Francesco Chirivì, Ester Maria Lucia Bevere, Lucia Tricarico, Michele D’Alto, Roberto Badagliacca, Natale D. Brunetti, Carmine Dario Vizza, Stefano Ghio

**Affiliations:** 1 Cardiothoracic Department, Policlinico Riuniti University Hospital, 71100 Foggia, Italy; lucia.tricarico.lt@gmail.com; 2 Department of Medical and Surgical Sciences, University of Foggia, 71100 Foggia, Italy; francesco.chirivi11@gmail.com (F.C.); estermarialuciabevere@gmail.com (E.M.L.B.); natalebrunetti@gmail.com (N.D.B.); 3 Department of Cardiology, A.O.R.N. dei Colli, Monaldi Hospital, University of Campania L. ‘Vanvitelli’, 80133 Naples, Italy; mic.dalto@tin.it; 4 Department of Clinical, Anesthesiological and Cardiovascular Sciences, I School of Medicine, Sapienza University of Rome, 00185 Rome, Italy; roberto.badagliacca@uniroma1.it (R.B.); dario.vizza@gmail.com (C.D.V.); 5 Division of Cardiology, Fondazione IRCCS Policlinico San Matteo, 27100 Pavia, Italy; s.ghio@smatteo.pv.it

**Keywords:** pulmonary hypertension, pulmonary arterial hypertension, endothelial dysfunction, flow-mediated dilatation

## Abstract

Pulmonary arterial hypertension is a complex pathology whose etiology is still not completely well clarified. The pathogenesis of pulmonary arterial hypertension involves different molecular mechanisms, with endothelial dysfunction playing a central role in disease progression. Both individual genetic predispositions and environmental factors seem to contribute to its onset. To further understand the complex relationship between endothelial and pulmonary hypertension and try to contribute to the development of future therapies, we report a comprehensive and updated review on endothelial function in pulmonary arterial hypertension.

## 1. Introduction

Pulmonary arterial hypertension (PAH) is a rare disease primarily affecting the pre-capillary pulmonary vascular bed. It is characterized by pulmonary artery remodeling (an obstructive remodeling of the pulmonary arterioles coupled with vascular pruning) and progressive increased pulmonary vascular resistance (PVR), increasing right ventricular afterload. Despite significant advancements in treatment, PAH remains a devastating disease leading to a progressive increase in pulmonary vascular resistance (PVR), subsequently resulting in right heart failure, hospitalization, and death [1].

The pathophysiology of PAH is complex, involving various molecular mechanisms, with endothelial dysfunction (ED) playing a pivotal role in disease progression.

However, a comprehensive understanding of the pathophysiological mechanisms underlying PAH is lacking.

Inflammation plays an important role in the onset and progression of PAH. Perivascular and interstitial inflammatory infiltrates have been identified in all pathological patterns of PAH [2], with elevated levels of chemokines and cytokines observed in PAH patients. Nevertheless, inflammation is likely not the sole contributor to disease development and progression.

The complex interplay among different signaling pathways such as nitric oxide, prostacyclin, serotonin, hormonal pathways, genetic predisposition, and environmental and inflammatory factors contributes to the intricate phenotype seen in patients with PAH. These interactions result in the pathological changes described in PAH.

The most common pathological characteristics include the dysfunction of pulmonary artery endothelial cells (PAECs), proliferation and migration of pulmonary artery smooth muscle cells (PASMCs), and dysregulated fibroblast activity [3]. These dynamic changes lead to a phenotype of dysregulated vasoconstriction, microthrombosis, vascular fibrosis, and pathogenic remodeling of pulmonary vessels [4].

PAH is characterized by local endothelium dysfunction, impaired synthesis of growth factors and cell survival, and imbalance between vasodilation and constriction [5].

ED plays a central role in the development and progression of PAH [6].

The glycocalyx is a gel-like layer covering the surfaces of endothelial cells, composed of proteoglycans, glycoproteins, and glycolipids. It plays a crucial role in endothelial function by regulating vascular permeability, inflammation, and vascular tone [7].

Additionally, the glycocalyx interacts with a wide variety of proteins and may contribute significantly to processes such as the regulation of vascular permeability, the modulation of local inflammatory processes, and the transmission of shear stress. The destruction of glycocalyx has been implicated in the development of pulmonary hypertension [8].

While research on PH has mainly focused on the study of pulmonary circulation, in recent years, the study of peripheral endothelial function in non-pulmonary vascular districts growth in interest. Non-invasive methods are available to assess endothelial function. However, it remains unclear whether endothelial function correlates with the hemodynamic pattern or can assist clinicians in predicting the pharmacological response to specific pulmonary vasodilators. Furthermore, it is not known whether there is a possibility of predicting adverse events through the evaluation of endothelial function.

To further understand the complex relationship between endothelial and pulmonary hypertension and try to contribute to the development of future therapies, we report a comprehensive and updated review on endothelial function in pulmonary arterial hypertension.

## 2. What Is Endothelial Function and What Regulates It?

The endothelium, a monolayer of cells lining the vasculature, performs a pivotal role in cardiovascular health, governing various functions critical for vascular balance. Compromised endothelial function has been associated with several pathologies, including pulmonary arterial hypertension (PAH).

This cellular layer encompasses arteries, veins, and capillaries, forming a continuous interface within the vascular system [9].

Among its primary functions, the endothelium actively modulates blood flow distribution by releasing vasoactive substances such as nitric oxide (NO) and prostaglandins and vasoconstrictive substances, such as endothelins, which modulate vessel tone and diameter [10].

Additionally, it functions as a selective barrier, controlling the exchange of molecules and cells between the blood and surrounding tissues, influencing vascular permeability.

The endothelium generates anticoagulant factors, which are necessary for avoiding thrombotic episodes and inhibiting the development of intravascular clots. In addition, it regulates inflammatory pathways and immunological responses, which are critical for protecting the body from infections and preserving vascular integrity [11].

Numerous factors have a significant impact on endothelial function. Shear stress and hemodynamic pressure cause the activation of endothelial NO synthase (eNOS), which modulates the vascular tone increasing NO availability [12].

Vasomotor activity, permeability, and inflammatory responses are all directly impacted by the complex modulation of endothelial function by endocrine factors such as insulin, adrenaline, and neurotransmitters [13].

Endothelial integrity is compromised by oxidative stress and chronic inflammation, which promote a prothrombotic state and contribute to the advancement of atherosclerosis [14].

In conclusion, it is important to understand the complex regulatory processes that include inflammatory, hormonal, hemodynamic, and lifestyle aspects to comprehend the range of diseases linked to impaired endothelial function, including PAH.

To further understand the complex relationship with endothelial and pulmonary hypertension (PH), more research on the pulmonary endothelium is currently being conducted.

## 3. Why Is Endothelial Function Relevant in PAH?

The pathological condition known as PAH is typified by a malfunctioning endothelium, which results in altered synthesis of growth factors, cell survival, and the balance between vasodilation and vasoconstriction.

Endothelial–mesenchymal transition in pulmonary endothelial cells (ECs) disrupts pulmonary vascular homeostasis, which, in turn, causes pulmonary artery vascular remodeling and a gradual rise in vascular resistance [5].

The endothelium maintains its genetic integrity and quiescence in normal circumstances. In order to restore equilibrium, it activates and releases growth factors and cytokines that stimulate the proliferation of endothelial, fibroblast, and smooth muscle cells. Furthermore, it regulates apoptosis, coagulation, the activation of inflammatory cells, and vasoreactivity [15]. 

When these things happen all at once, they cause arterial occlusion and muscularization, which result in vascular lesions.

One of the main pathogenetic components of PAH is thought to be endothelial cell injury, which starts poorly known cellular signaling cascades [16,17].

An ED condition is brought on by prolonged or chronic endothelium activity and is defined by the loss of endothelial homeostatic activities.

Numerous variables, including cilia length, ionic channel function modification, shear stress, hypoxia, inflammation, and genetic and epigenetic factors, have been suggested in the literature as possible initiators of ED in PAH [18,19].

Vasoconstrictor factors like endothelin-1 (ET-1) [20] and thromboxane [21] and proliferative factors like vascular endothelial growth factor (VEGF), fibroblast growth factor 2 (FGF2) [22], and C-X-C motif ligand 12 (CXCL12) [23] are produced when the endothelium moves from a quiescent to a hyperactive state.

Moreover, the synthesis of vasodilators such as prostacyclin and NO decreases in a chronically active state [24].

An increasing body of evidence strongly suggests that endothelial function can be regarded as both a prognostic and diagnostic parameter for PH. Currently, several molecular pathways involved in the pathogenesis of PAH are well documented [18]. In recent years, pharmaceutical interventions targeting altered molecular pathways have been developed for treatment. The primary therapy focus is on reinstating proper endothelial function to achieve improvements in prognosis and quality of life.

Another mechanism explored for PAH therapy is the endothelin pathway.

ET-1 binds to endothelin receptors A and B (ETA, ETB) on PASMCs, causing an increase in intracellular calcium due to inositol triphosphate and vasoconstriction via phospholipase C-B. Moreover, ET-1 has been shown to trigger fibrogenesis, which has been linked to PAH through its effects on matrix metalloproteinases and smooth muscle hypertrophy, which happen in tandem with inflammatory mediators, hormonal agents, oxidative stress, and hypoxia [25].

Another involved pathway is that of prostanoids.

Lipids contained in the plasma membrane are the source of prostanoids. These lipids change into 5-, 8-, 11-, and 14-eicosatetraenoic acid or arachidonic acid when phospholipase A2 acts upon them. Arachidonic acid is further processed to produce leukotrienes (LT), prostaglandins (PG), and thromboxane (TXA2). Arachidonic acid is then transformed by COX into TXA2 and prostacyclin (PGI2).

PGI2 functions as a vasodilator, widening blood vessels and preventing platelet aggregation, while TXA2 is a potent drug that constricts blood vessels and causes platelets to congregate [26].

Theory suggests that decreased PGI2 and elevated TXA2 may be responsible for the constrictive aspect of PAH. This theory is supported by observational evidence, which shows that patients with PAH have lower PGI2 synthase mRNA levels in their pulmonary vessels and that animal models that induce PAH through hypoxia have higher TXA2 levels and sensitivity. Observational studies have shown also that patients with PAH have higher levels of TXA2 metabolites and lower excretion of PG metabolites in their urine than healthy individuals [21,27,28].

Vascular shear stress, inflammation, hypoxia, and genetic variants in TGF-β receptors contribute to triggering the activation of COX-2 [29].

Trying to correct ED involves more than targeting vasodilation and/or vasoconstriction processes alone. It means adopting a comprehensive approach that includes the metabolic, hormonal, and inflammatory functions of the endothelium. Scientific evidence indicates that enhancing endothelial function can potentially slow disease progression and improve symptoms. This emphasizes the crucial need for in-depth research on endothelial function and prognostic implications of its corrections on patients’ lives.

### Genetics

The pathogenesis of PAH involves different molecular mechanisms, with ED playing a central role in disease progression. Both individual genetic predispositions and environmental factors seem to contribute to its onset [30].

Understanding the genetic mutations responsible for ED is crucial for discovering the molecular basis of the disease and identifying novel therapeutic targets that can help to improve the prognoses of patients. In this part of our review, we aim to explore the main genetic alterations underlying ED in PH.

## 4. Genes in PAH

**Figure 1:**
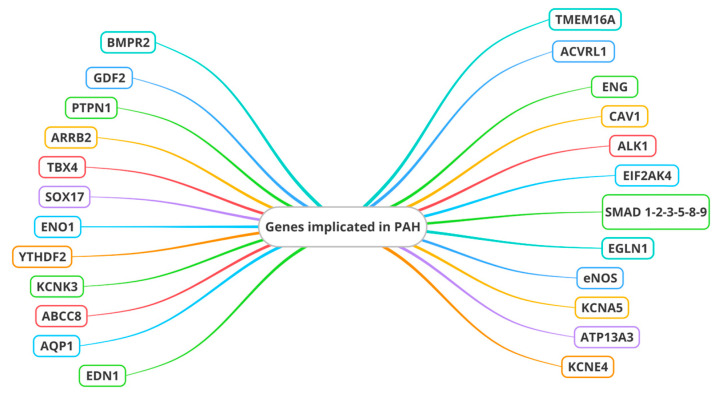
Genes involved in endothelial dysfunction in pulmonary arterial hypertension. *BMPR2: Type II receptor of bone morphogenetic protein; ACVRL1: activin receptor type 1; ENG: endoglin; CAV1: Caveolin 1; ALK1: Anaplastic lymphoma receptor tyrosine kinase 1; KCNK3: Potassium Two Pore Domain Channel Subfamily K Member 3; EIF2AK4: Eukaryotic Translation Initiation Factor 2 Alpha Kinase 4; SMAD: Mothers Against Decapentaplegic Homologs; TBX4: T-box transcription factor 4; SOX17: SRY-box transcription factor 17; ENO1: Alpha-enolase 1; EGLN1: Egl-9 family hypoxia inducible factor 1; eNOS: Endothelial nitric oxide synthase; KCNA5: Potassium voltage-gated channel subfamily A member 5; ABCC8: ATP-binding cassette subfamily member 8; ATP13A3: ATPase 13A3; AQP1: Aquaporin 1; KCNE4: Potassium Voltage-Gated Channel Subfamily E Regulatory Subunit 4; TMEM16A: Transmembrane Protein 16A; EDN1: Endothelin 1; ARRB2: Beta-arrestin 2; PTPN1: Protein tyrosine phosphatase non-receptor type 1; GDF2: Grow Differentiation Factor 2; YTHDF2: YTH N6-methyladenosine RNA binding protein 2*.

*Type II receptor of bone morphogenetic protein (BMPR2)* is the main gene associated with the TGF-β receptor superfamily that is implicated in PAH. Other genes associated with the TGF-β receptor superfamily include the genes encoding *activin receptor type 1 (ACVRL1), endoglin (ENG), Caveolin 1 (CAV1), anaplastic lymphoma receptor tyrosine kinase 1 (ALK1), Mothers Against Decapentaplegic Homologs (SMAD) 8 (or SMAD9), and SMAD4* [31] (Figure 1).

The TGF-β family regulates cell proliferation, differentiation, and apoptosis, as well as the process of mesenchymal-to-endothelial transition [32]. The TGF-β–ACVRL1–ENG cascade activates the phosphorylation of SMAD 1/5/8 and SMAD2/3 through receptors such as BMPR2, inducing the expression of growth factors such as fibroblast growth factor 2 (FGF2) and platelet-derived growth factor 2. Therefore, TGF-β also determines the induction of smooth muscle cell proliferation [33].

BMPR2 is a transmembrane serine/threonine receptor kinase encoded by the *BMPR2* gene [34]. This receptor interacts with bone morphogenetic proteins (BMPs), members of the TGF-beta ligand superfamily involved in paracrine signaling. BMPs play a crucial role in various cellular functions, including osteogenesis, apoptosis, cell growth, and differentiation. BMPR2 is important in promoting the survival of pulmonary arterial endothelial cells (PAECs), and it has an anti-proliferative effect on pulmonary arterial smooth muscle cells (PASMCs) [35,36,37,38,39,40,41]. The BMP signaling pathway initiates with the binding of a BMP to the Type II receptor, followed by the recruitment of a Type I receptor and the activation of an R-SMAD, a transcriptional regulator.

In the context of PAH, over 380 mutations in the *BMPR2 gene* have been identified, especially those of the loss-of-function type [42,43]. It is relevant that the low penetrance of PAH development in individuals with BMPR2 mutations has also been observed in experimental models of PH, suggesting the existence of genetic or environmental factors influencing BMPR2-dependent signaling [44,45,46,47,48,49,50]. The reduced presence of BMPR2 in pulmonary ECs in PH suggests a significant role for BMPR2 mutations in ED in PAH [47,48,49,50]. Furthermore, the correlation between endothelial BMPR2 expression levels and the development of PAH is supported by the observation that mice with specific *BMPR2 gene* silencing in ECs were more susceptible to developing PAH [51,52]. Mutations identified in the *Growth Differentiation Factor 2 (GDF2) gene*, which codes for the BMP9 ligand, have been observed in PAH patients, associated with reduced levels of BMP9 and BMP10 in the blood. The overexpression of BMP9 has selectively enhanced BMPR2 signaling in PAECs, reversing PH in animal models [53]. These findings suggest a causal role for these mutations in EC dysfunction and the subsequent development of PAH. Ali MK et al. established that PTPN1 (PTP1B, protein tyrosine phosphatase non-receptor type 1) is a modulator of BMPR2 signaling within PAEC. In patients affected by PAH, there is a down-regulation of PTPN1, and this decrease is associated with impaired endothelial function in PAEC [54]. A study led by Wang et al. revealed that the depletion of beta-arrestin 2 (ARRB2) in PASMC with reduced BMPR2 restored regular signaling, reversed diminished contractility, and mitigated the increased proliferation. Furthermore, in mice experiencing the inducible loss of BMPR2 in smooth muscle cells (SMC), the reduction in ARRB2 effectively prevented the persistence of PH [55]. Therefore, dysfunction in BMPR2 signaling disrupts the delicate balance between endothelial cell proliferation and apoptosis, contributing to vascular remodeling [56]. 

Members of the *SMAD* family are crucial mediators of transforming growth factor-beta (TGF-β) signaling, which plays a central role in vascular remodeling. Aberrant TGF-β signaling, often associated with mutations in *SMAD* genes, contributes to ED in PAH [57].

*CAV1* is located in the endothelia of pulmonary arteries [58]. This gene encodes a membrane protein localized on the cell surface caveolae, regulating signaling for TGF-β, NO, and G proteins. BMPR2 is also located in caveolae and directly interacts with CAV1 in vascular smooth muscle cells [59,60].

These represent rare causes and should be investigated if BMPR2 mutations are not detected. 

Other rarer mutations have been described. Among them, there are the following:

- *Transcription factors like T-box transcription factor 4 (TBX4)* and *SRY-box transcription factor 17 (SOX17)* [61,62,63].
- *Alpha-enolase 1 (ENO1)*, which influences genes associated with mitochondria and the PI3K-Akt signaling pathway [64];
- *YTH N6-methyladenosine RNA binding protein 2 (YTHDF2)*, which increased pulmonary vascular resistance, right ventricular hypertrophy, macrophage polarization, and oxidative stress [65].
- *Egl-9 family hypoxia inducible factor 1 (EGLN1)* involved in the remodeling of pulmonary vasculature and development of obliterative pulmonary vascular lesions, contributing to the process of endothelial-to-mesenchymal transition (EndoMT) [66,67,68,69,70,71].
- Endothelial nitric oxide synthase (*eNOS*), crucial for NO synthesis. Impaired *eNOS* activity is a hallmark of ED in PAH, leading to reduced NO bioavailability and enhanced vasoconstriction. Genetic variations affecting *eNOS* function have an important impact on endothelial homeostasis and the development of PAH [72].
- Channel genes. Mutations in the Potassium Two Pore Domain Channel Subfamily K Member 3 (*KCNK3*) gene, encoding an outward-rectifying potassium channel, are related to ED and, thus, to pulmonary vascular remodeling. Another member of the potassium channel family, namely potassium voltage-gated channel subfamily A member 5 (*KCNA5*), plays a role in the ED of PH due to its regulatory function in pulmonary vascular tone, cell proliferation, apoptosis, and oxygen sensitivity [73,74]. Two other channel genes are involved: *ATP-binding cassette subfamily member 8* (*ABCC8*) and *ATPase 13A3* (*ATP13A3*). Another channel gene, *aquaporin 1* (*AQP1*), was reported in one study conducted by Stefan Graf et al. [41]. *ABCC8* is expressed in PAECs and PASMCs [75,76], influencing vasoreactivity and cell proliferation, thereby impacting pulmonary vascular tone and remodeling. *KCNE4* (Potassium Voltage-Gated Channel Subfamily E Regulatory Subunit 4) also seems to be involved in vascular tone regulation in pulmonary arteries [77]. In the pathogenesis of PAH, Cl-activated Ca^2+^ channels (CaCCs) also appear to be implicated. It has been demonstrated that the most significant member of the CaCCs, Transmembrane Protein 16A (TMEM16A), contributes to the pathogenesis of IPAH in PASMCs. It was proposed that the extracellular signal-regulated kinase ½ (ERK1/2) pathway is specifically influenced by elevated *TMEM16A* activity, leading to functional consequences such as reduced NO production, alterations in Ca^2+^ dynamics and eNOS activity, the proliferation of PAECs, wound healing, tube formation, and the acetylcholine-mediated relaxation of human pulmonary arteries [78].
- ET-1 (endothelin 1) is a potent vasoconstrictor. Genetic variations in the *EDN1* gene contribute to ED by promoting excessive vasoconstriction and smooth muscle cell proliferation. So, *EDN1* has an important impact on the delicate balance of endothelial homeostasis [79].
- *Eukaryotic translation initiation factor 2-alpha kinase 4 (EIF2AK4).* This gene is involved in the regulation of protein synthesis in eukaryotic cells. Its specific functions can vary depending on the cellular context and environmental signals. *EIF2AK4* plays an important role in the cellular stress response, particularly in endoplasmic reticulum (ER) stress. When cells undergo stress, such as hypoxia or the accumulation of misfolded proteins in the endoplasmic reticulum, *EIF2AK4* can be activated. Once activated, it phosphorylates a subunit of the protein translation initiator, called eIF2α. This phosphorylation prevents the initiation of global protein translation, allowing cells to adapt to stress by activating survival or repair programs [80,81] (Table 1).

The availability of genetic–molecular diagnostics represents an innovative advancement in patient care, encompassing genetic counseling for PAH [82]. BMPR2 mutations have been identified in a significant proportion of familial and idiopathic PAH cases. For this reason, counseling and screening for BMPR2 mutations are often recommended not only for individuals with idiopathic PAH (IPAH) but also in sporadic and induced-by-anorexigens forms, as well as those with a family history of PAH.

In cases where BMPR2 mutations are not detected in familial PAH or IPAH patients under 40, or when PAH is identified in individuals with a personal or family history of hereditary hemorrhagic telangiectasia, screening for Activin A Receptor Like Type 1 (ACVRL1) and endoglin (ENG) genes may be recommended. If no mutations are found in BMPR2, ACVRL1, and ENG genes, screening for rare mutations (e.g., KCNK3, CAV1) becomes a viable option [83]. As well as variations in BMPR2, mutations in related genes within the BMPR2 signaling pathway, including ACVRL1, ENG, SMAD9, and GDF2, are sometimes responsible for sporadic IPAH. Such variations are infrequently observed in PAH associated with other diseases (APAH) or caused by medication/toxin exposure. 

Patients with sporadic or familial pulmonary veno-occlusive disease/pulmonary capillary hemangiomatosis (PVOD/PCH) may be subjected to screening for eukaryotic translation initiation factor 2-alpha kinase 4 (EIF2AK4) mutations. The identification of a bi-allelic EIF2AK4 mutation allows us to confirm the diagnosis of PVOD/PCH, eliminating the need of a lung biopsy for histological verification. In hereditary hemorrhagic telangiectasia (HHT), mutations in ENG or ACVRL1 are frequently observed, while SMAD4 mutations affect a small percentage of patients. Mutation in the gene encoding GDF2/BMP9 is also an extremely rare genetic cause of HHT [84].

## 5. Aspects of Vascular Biology

The disruption of pulmonary vascular homeostasis and subsequent vascular remodeling are associated with the EndoMT that occurs in ECs. This aberrant remodeling of the distal pulmonary arteries, which results in elevated vascular resistance, is what defines PAH. The proliferation of PASMCs and PAECs is a characteristic of this process, which entails modifications to vasoconstriction mechanisms [85]. Significant progress has been made in identifying the mechanisms behind dysregulated pulmonary vascular tone in preclinical animal models and human PH [86]. Vasodilatory therapies have benefited greatly from this improved understanding. These therapies show varying degrees of efficacy in PH patients’ symptom relief and life extension.

Furthermore, fresh understanding of the pathophysiology underlying the genesis of the distinctive lesions of PH has been provided by contemporary molecular biology research. Together with complicated lesions, plexiform lesions comprise a complex component of pulmonary arterial pathology. This includes different structures such as onion-skin lesions, plexiform core lesions, and dilation lesions, which are often seen in a closely connected topographic array. However, further research is needed to completely understand the underlying pathophysiological significance of these prevalent vascular changes in PAH.

According to recent research, systemic blood vessels that pass through the pulmonary artery adventitia or are located in the peribronchial connective tissue, such as the vasa vasorum and bronchial arteries, may play a role in the development of plexiform vasculopathy [87].

The idea of vascular shunting is based on the examination of successive sections from patients with PAH by utilizing digital three-dimensional reconstruction. According to this theory, plexiform lesions serve as networks that connect bronchial microvessels to the pulmonary arterial and venous systems [88].

A morphometric analysis of lung tissue sections from patients with PAH, including cases of idiopathic PAH (IPAH) and heritable PAH (HPAH) associated with mutations in the BMPR2 gene, has revealed the presence of vascular shunts between bronchial and pulmonary blood vessels [89].

The remodeling of the pulmonary veins has been linked to bronchial artery enlargement and expansion, as well as an increase in the number of bronchial microvessels in people with BMPR2 mutations [90].

Furthermore, large fibrovascular structures, known as “SiMFis” (single millimetric fibrovascular lesions), appear to act as conduits between pulmonary arteries and veins and the systemic circulation.

It is currently unknown if this expanded systemic vasculature in PAH can avoid pulmonary artery blockage at the outset [87]. The identification of disruptions in several cellular and molecular systems underscores the complex etiology of the illness. Growth factors, cytokines, metabolic signals, elastases, and proteases are a few of these activities.

Notably, recent studies suggest that stem and progenitor cells may have an impact on the disease’s course, as well as its management [91]. These cells can promote the development of new blood arteries and help to repair a damaged endothelium, according to experimental research. Stem-cell-based treatments for PAH are now the subject of intensive research [91]. The therapeutic potential of stem/progenitor cell therapies in PAH has been evaluated through both experimental studies and clinical assessments [92]. Data derived via the meta-analysis of 28 preclinical studies that used stem/progenitor cell treatments (obtained from human or rat bone marrow, adipose tissue, or human umbilical cord blood mesenchymal cells) showed that therapies using stem/progenitor cells were associated with significant improvements in pulmonary hemodynamics in animal models of PAH when compared to control treatments [93].

Apoptosis resistance, inflammation, and aberrant vascular proliferation are only a few of the pathogenetic features of PAH that have been connected in recent years to aberrant microRNA (miRNA) expression.

Small non-coding RNA molecules known as miRNAs attach to the 3′UTR sections of target mRNAs to post-transcriptionally control gene expression by either degrading the mRNAs or inhibiting translation.

Numerous investigations have revealed that some miRNAs are dysregulated in PAH patients and animal models, indicating possible functions as biomarkers and targets for treatment. MiR-204 is one of the miRNAs linked to PAH that is dramatically down-regulated in the lungs of both PAH patients and rats with PH induced.

Reintroducing miR-204 in human pulmonary ECs and mouse models has been demonstrated to enhance hemodynamic parameters and reduce the proliferation of pulmonary vascular smooth muscle cells. MiR-21 is another miRNA that is relevant to PAH; it is up-regulated in rat models, as well as in the pulmonary tissues of PAH patients [94]. Through the suppression of BMPR2, a protective factor in the pathophysiology of PAH, MiR-21 encourages the proliferation and survival of smooth muscle cells in the pulmonary vascular system [95].

Other possibilities, such miR-22 and the miR-17/92 cluster, have also been identified using miRNA expression profiling as potential contributors to the intricate signaling network that underlies the pathophysiology of PAH.

These miRNAs suggest a wide range of biological consequences in PAH by regulating many important pathways, including the TGF-β, ETS-1, and reactive oxygen species pathways. Despite progress in comprehending the function of miRNAs in PAH, their practical applications are still difficult.

The stability of miRNAs, tissue-specific targeting, and more efficient delivery are among the challenges. To overcome these restrictions, creative solutions are starting to appear, such as the use of viral vectors and nanoparticles.

New insights into the vascular biology of PAH are revolutionizing our knowledge and strategies for managing this incurable illness. A number of molecular and cellular pathways offer new therapeutic and diagnostic strategies in PAH.

Prostacyclin analogs are synthetic forms of prostacyclin, an antithrombotic, anti-proliferative, vasodilatory, and anti-inflammatory agent generated by the vascular endothelium [96].

An analog of the prostacyclin receptor called selexipag was released a few years ago [97].

Lung tissue, kidneys, and the colon contain ET-1, a naturally occurring peptide that is recognized for its capacity to narrow blood channels. It is released by vascular ECs. Patients with PAH have higher levels of ET-1, which primarily acts via the endothelin receptor A (ETA) to cause cellular proliferation and pulmonary artery constriction. Thus, one of the most important strategies for the treatment of PAH is to disrupt ET-1/ETA receptor connections. By activating endothelial ETB receptors, ET-1 can also reduce the constrictive effects of its own activities by inducing the production of vasodilators such as NO [98].

In particular, advances in understanding the sGC-cGMP pathway of NO reveal novel treatment opportunities. Strongly acting as a vasodilator, endothelial NO promotes the production of cGMP. Its dysfunction has been observed in patients with PAH, resulting in lower plasma NO [99].

Additionally, research has indicated that eNOS may uncouple, causing the production of reactive oxygen species rather than NO and worsening vascular disease.

Optimizing the usage of sGC stimulators, PDE-5 inhibitors, and NO donors might lead to selective pulmonary vasodilation and a decrease in right ventricular hypertrophy.

The goal of RNA manipulation, which includes the use of miRNA mimics or antagomirs and small interfering RNAs (siRNAs), is to more precisely regulate gene expression. Given the significance of angiogenesis and the preservation of microvasculature, several models of PAH are being treated with angiogenic growth factors or angiogenesis inhibitors [100]. Modifying angiogenesis in the pulmonary vascular bed may restore the proper balance between vascular breakdown and regeneration [101].

Repairing or replacing damaged pulmonary vascular tissue is today a possibility thanks to stem cells and regenerative medicines. In addition, mitochondrial dysfunction and cellular metabolism play important roles in PAH, showing the possibility of using them as potential therapeutic targets, enhancing cellular bioenergetics and decreasing aberrant proliferation using mitochondrial metabolism modulators [102,103].

The pathophysiology of PAH is also closely linked to innate immunity and inflammasome pathways. Targeting these immune responses through therapeutic interventions holds promise for more precise treatments focusing on the inflammatory aspects of the disease. The application of pharmacogenomic analysis offers a personalized approach to maximize therapeutic efficacy and minimize adverse effects by tailoring treatments based on individual genetic traits. This approach is particularly valuable in managing PAH due to the substantial variability in patients’ responses to therapies.

Future studies of vascular biology in PAH will concentrate on fusing these fresh viewpoints with tried-and-true treatment modalities. The ultimate objective is to create more individualized and efficient treatment plans that can greatly enhance the prognosis and quality of life for PAH patients [104] (Table 2).

### How to Evaluate Peripheral Endothelial Function in Patients with Pulmonary Hypertension

A growing amount of research is indicating that peripheral and pulmonary endothelium play roles in the development of a number of cardiovascular diseases, including PH.

Numerous studies conducted on endothelial function have investigated the peripheral participation of microcirculation (ungual capillaries) and the macrocirculation (brachial and radial arteries). Angiography and ultrasonography can be used to evaluate major arteries and characterize and quantify their vasodilatory activity. Reduced vasodilation or a vasoconstrictive reaction in response to vasodilatory chemicals produced by the endothelium after internal stimuli are symptoms of ED [37].

There are three primary methods for evaluating endothelial function: in vitro, ex vivo, and in vivo.

An established method for assessing ED valuating vascular tone is endothelium-dependent dilation (EDD). Reactive hyperemia, increased blood flow after ischemia, and arterial blockage are indicators used to evaluate vascular tone. Blood flow parameters such as peak blood flow before and after an ischemia challenge may be measured in a variety of vascular locations [105,106,107].

EDD may be measured in vivo by utilizing methods such as intra-arterial infusion, which involves the delivery of acetylcholine through the brachial artery. This substance is an endothelium-dependent vasodilator that causes EDD, which is measurable via ultrasound imaging [108]. In a similar manner, other NO-enhancing substances including bradykinin and methacholine might be used [109]. Furthermore, the introduction of different vasoactive agents can be used to assess endothelial reactions, offering information on possible ED.

Endothelial function is also studied using intravenous infusion via the dorsal hand vein approach [110,111]. This entails sticking a needle into the vein on the back of the hand, then measuring the vein’s diameter using a linear transformer after the vasoactive drugs are administered. These drugs can cause a variety of physiological reactions, not all of which are connected to EDD. Although these intravenous and intra-arterial infusion techniques are more time-consuming and intrusive than alternative non-invasive approaches, they are generally safe [109,110,111].

FMD is the most useful technique for evaluating endothelial function in vivo. This method is simple, non-invasive, patient-safe, and cost-free. A physiological process known as “flow-mediated dilatation” takes place when blood flow through an artery increases and the vascular endothelium experiences a brief increase in shear stress. ECs release NO, a potent vasodilator, in response to this shear stress. Following its diffusion into the artery wall’s smooth muscle cells, NO raises cGMP, which, in turn, stimulates the smooth muscle cells to relax. To assess endothelial function, this dilation may be assessed using ultrasonography, usually in the brachial artery [112].

When assessing FMD, ultrasound and magnetic resonance imaging are essential tools for gathering information on vascular compliance via measures such as the augmentation index [113,114].

The brachial artery or, less commonly, the radial artery’s endothelial function is evaluated using ultrasonography in the context of FMD [38,115]. FMD is the term used to describe the capacity of blood arteries to expand in response to elevated flow or, more specifically, shear stress. To minimize sources of variability related to acquisition, processing, and interpretation, the Brachial Artery Reactivity Task Force established recommendations in 2002 [116]. It is simple to perform an ultrasonography examination of peripheral endothelial function using the FMD technique: the baseline artery diameter is measured and the sphygmomanometer cuff is positioned. The cuff is inflated, held in place for a minimum of five minutes, and then released. The diameter of the studied artery is then measured. Ultimately, measures like FMD% may be obtained by calculating the ratio, either as a percentage or by multiplying by 100, between the change in the baseline diameter of the examined artery and the baseline diameter of the artery under evaluation [117].

Furthermore, low-flow-mediated constriction (L-FMC) provides additional information on ED. Vascular reactivity is shown by L-FMC, which measures the variation in arterial diameter and flow during the last stage of arterial occlusion. Rather than only representing EDD, the resulting L-FMC values show the combined effect of relaxing and contracting components originating from the endothelium. The measure is expressed as the proportion of the artery diameter that is reduced after occlusion in comparison to the resting condition [118,119]. Reactive hyperemia has been expanded to the microvascular level, where the reactive hyperemia index (RHI) [120] is used to measure it. Peripheral arterial tone or digital pulse waveforms are used to measure EDD. To evaluate pulse wave forms before and after brachial artery closure, disposable plethysmographic probes are placed on the index fingers [121]. These pre- and post-occlusion values are used to determine the RHI. A linear link between RHI and FMD has been identified in studies, and a correlation between RHI and cardiovascular events has also been demonstrated [122].

Venous occlusion plethysmography is a time-consuming, invasive procedure that requires a high degree of operator skill and graded intra-arterial injections of nitroprusside (to test endothelium-dependent vasodilation) and acetylcholine (to assess endothelial function). A cuff inflated to a pressure below diastolic levels is used to restrict venous return in venous occlusion plethysmography. This stops venous outflow and permits arterial blood to enter the limb. Based on changes in limb volume over a predetermined period of time, the measurement represents blood flow, which is impacted by vascular tone and resistance. This technique is used to measure local vascular tone after an ischemic event in an indirect manner. Changes in limb volume can be measured using a variety of means, such as air, water, and mercury, as well as impedance techniques [123,124,125,126].

This traditional method has been improved by adding automated procedures and using intra-arterial drug infusion concurrently. Variables like the length of cuff inflation can have a substantial influence on blood flow measurements, even if they are repeatable and reproducible [127].

Vascular permeability is measured via venous occlusion plethysmography, which also computes the capillary filtration coefficient (CFC), a permeability and blood vessel conductance measure [128,129].

Despite some early difficulties, this method distinguishes between variations in blood volume caused by capillary permeability and vascular occlusion [130,131]. Venous occlusion plethysmography, in contrast to post-occlusive reactive hyperemia (PORH) measurement, uses gradual increases in cuff pressure, enabling limb volume changes to represent fluid flow from microvasculature. Careful recording of measurement locations and cuff pressure increments is necessary to ensure repeatability [132,133].

The endothelium secretes tissue plasminogen activator (tPA), which inhibits the growth of atheroma, platelet activation, and thrombus formation [134]. Venous occlusion plethysmography is typically used in conjunction with tPA release measurement [135,136,137].

Novel techniques such as enclosed zone FMD (ezFMD) have been proposed to overcome some shortcomings of conventional FMD techniques. By measuring oscillations in the intra-arterial pressure’s amplitude, ezFMD is able to evaluate variations in vascular volume [138]. The evaluation of arterial dilatation in response to ischemia, which may be a sign of impaired lower-limb endothelial function, is made possible by this approach.

Another non-invasive technique used to measure EDD in the microvasculature of the skin in response to different stimuli is laser Doppler flowmetry (LDF) [139]. With this method, a laser beam is injected into the skin and soft tissue, and some of the light is reflected back by the red blood cell movement. Using the Fizeau Doppler principle [140], the blood flow velocity is then inferred from the frequency shift of this backscattered light. Laser Doppler imaging (LDI) or perfusion imaging (LDPI) was created with the help of technology, which increased the method’s repeatability and allowed for a wider volume to be investigated [141]. The skin microvasculature’s endothelium can be stimulated via a number of methods, such as iontophoresis, PORH, localized thermal hyperemia, or transdermal delivery of acetylcholine, as well as other medications via microdialysis [142,143]. Clinical uses for these laser-based techniques have also been identified in the assessment of microcirculation, particularly in the investigation of burns and wounds [144].

Doppler echocardiography, magnetic resonance imaging, and positron emission tomography are further non-invasive methods for assessing endothelial function. While MRI offers better picture quality than ultrasound, it is more expensive and has less temporal resolution. New sophisticated techniques for studying peripheral microcirculation are nailfold videocapillaroscopy (NVC) and near-infrared spectroscopy (NIRS) [145].

Apart from EDD, other methods used to evaluate vascular tone modulation are assessing arterial stiffness or compliance and examining the features of pulse waves [146]. An important determinant of arterial wall stiffness is pulse wave velocity, which is the rate at which the pressure wave travels through the arterial tree. Pulse wave velocity is inversely correlated with arterial stiffness and may be evaluated at locations such as the brachial artery, carotid artery, or aorta. The primary measure of stiffness in the main arteries is the carotid–femoral pulse wave velocity [147]. Techniques including oscillometric approaches and one-point pulse wave velocity have been presented [148,149] to expedite arterial compliance measurement.

Pulse wave velocity is not frequently used in clinical settings primarily because it is not practicable in daily practice [150]. The true advantage of such alterations in a therapeutic environment has not yet been thoroughly shown, despite drugs like angiotensin-converting enzyme inhibitors and angiotensin receptor blockers being known to lower pulse wave velocity [151,152] (Table 3).

Measuring endothelial function through glycocalyx thickness involves assessing the thickness of this layer as an indicator of endothelial health and function, provides valuable insights into vascular integrity, and may be used as a biomarker of cardiovascular diseases, such PH [7]. Several techniques can be used to measure glycocalyx thickness (intravital microscopy; sidestream dark-field (SDF) imaging; two-photon microscopy; biochemical assays; clinical assessment).

## 6. Endothelial Function and Pulmonary Therapy

The pathophysiology of PH is significantly influenced by endothelial function, which may also help to distinguish between pre-capillary and post-capillary forms of the disease. Excessive vasoconstriction is a sign of ED since it is caused by a decrease in prostaglandin and NO availability, as well as an increase in thromboxane A2 [154].

The NO pathway, prostacyclins and prostanoids, and endothelin receptor blockers of ET-1 are just a few of the molecular pathways involved in the complex pathophysiological course of PAH [155].

For instance, the identification of abnormalities in the synthesis of endothelium-originated vasoregulatory agents, such as NO, prostacyclin (PGI2), and ET-1, has led to the development of therapeutic applications for drugs like ET-1 receptor blockers, phosphodiesterase-5 (PDE5) inhibitors, cGMP stimulators, and PGI2 analogs [156,157,158,159,160].

There are several medications that aim to correct endothelial function approved by the FDA for the treatment of PAH [161]. In PH, therapeutic approaches that simultaneously target several endothelial pathways aim to enhance the vasodilatory response of the circulatory system [162,163,164].

Prostacyclin analogs and the analog of the prostacyclin receptor are used to mimic the prostacyclin action [165].

Synthetic prostanoids, which include epoprostenol and treprostinil administered intravenously or subcutaneously, iloprost and treprostinil inhaled, and treprostinil and beraprost taken orally, have a well-established role in the treatment of PAH [166].

A well-known prostacyclin is epoprostenol, which has been shown to increase functional class (FC) and incremental exercise capacity (6MWD) and lower the death rate [167].

These medications have been demonstrated to enhance cardiopulmonary hemodynamics, as indicated by the decline in right atrial pressure, pulmonary vascular resistance, and mean pulmonary arterial pressure (mPAP) [168].

Furthermore, selexipag has shown promise in the management of PAH. Selexipag at a dose of 1600 µg twice daily or a placebo was randomly assigned to 1156 PAH patients in a phase 3 randomized double-blind, placebo-controlled trial conducted by Sitbon and colleagues [97]. The group treated with selexipag had a lower risk of mortality from all causes or complications connected to PAH. These data support the theory that patients with PH may have improved prognosis and quality of life if endothelial function is improved.

To counter the increased endothelin 1 levels, the medical community has been using bosentan, macitentan, and ambrisentan (FDA-approved endothelin receptor antagonists (ERAs) [98]) that inhibit endothelin receptors. Two novel biological treatments that specifically target ETA receptors are being studied: getagozumab, an antibody, and ETR-002 peptide, a treatment that resembles a vaccination [169].

NO is an endogenous vasodilator produced by the pulmonary endothelium through eNOS. The presence of NO then activates guanylate cyclase, leading to the production of cGMP. This, in turn, induces the relaxation of smooth muscle cells and, consequently, vasodilation. Moreover, cGMP regulates cellular proliferation, apoptosis, and inflammation. Eventually, cGMP is degraded by PDE-5 [153].

Drugs that target the NO pathway have been developed. Examples of these include soluble guanylate cyclase stimulators like riociguat and PDE-5 inhibitors like sildenafil and tadalafil. PDE-5 inhibitors raise cGMP levels, which improve heart function and vasodilation, as shown by improvements in the mPAP and 6-min walk test (6MWD).

Riociguat functions as a soluble guanylate cyclase (sGC) stimulant without requiring NO. Additionally, the sensitivity of sGC to NO is increased by this category of drugs, and PAH may generate less or inadequate amounts of NO. It has demonstrated improvements in 6MWD and mPAP, making it a critical treatment option for individuals who are not responding to PDE-5 inhibitors [99,170].

PDE-5 inhibitors have demonstrated improvements in pulmonary hemodynamics, exercise capacity, and symptoms among PAH patients, even including individuals with connective tissue diseases [171].

Germ-line mutations in the bone morphogenetic protein type II receptor (BMPR2; BMPR-II) gene cause the majority of cases of heritable pulmonary arterial hypertension (PAH). The BMPR2 receptor is a serotonin/threonine kinase receptor of the transforming growth factor-B (TGF-β) superfamily that activates different signaling cascades. Many of these cascades occur through either SMAD 2/3 (TGF-β) or SMAD 1/5/8 (BMP)

Even in the absence of BMPR2 mutations, increased transforming growth factor (TGF)β receptor signaling and decreased BMPRII signaling have been shown to contribute to PAH pathogenesis. Recently, research has focused on the potential mechanisms via which the imbalance of BMP/TGFβ signaling contributes to endothelial dysfunction, vascular remodeling, inflammation and disordered angiogenesis in PAH [172].

The loss of signaling results in greater TGF-β activity, promoting both proliferative and anti-apoptotic responses in PAECs and PASMCs, along with increased production of inflammatory cytokines. Increased TGF-B signaling decreases BMP signaling and vice versa, thus creating an imbalance and the over-promotion of TGF-β, leading to endothelial dysfunction and inflammation [173].

Sotatercept (ActRIIa-Fc) utilizes a TGF-β ligand trap to inhibit TGF-β activity and rebalance BMPR2 activity.

To improve the prognoses of PAH patients, research on more trustworthy indicators is still necessary [174].

Since the majority of PH treatments target endothelium components and processes, less intrusive, more affordable, and patient-acceptable follow-up might be achieved by utilizing methods like FMD for therapy response evaluation.

Patients with IPAH had reductions in PAP and PVR after inhaling iloprost; further analyses revealed a correlation between these reductions and FMD values [175].

A rapid and sustained improvement in endothelial function was observed after 4 weeks of bosentan therapy in patients with systemic sclerosis, a pathological condition that predisposes individuals to the development of PH. This improvement was demonstrated by an increase in brachial artery FMD%. In the prospective study by Sfikakis et al., it seemed to be associated with increased generation of NO and a subsequent decrease in FMD% at the brachial artery level. This improvement appeared to be linked to a drop in endothelin levels, similar to what has been seen in PH [176].

Hirashiki et al. looked at how bosentan altered peripheral endothelial function in PAH patients as opposed to those with chronic thromboembolic pulmonary hypertension (CTEPH). In this study, patients with inoperable CTEPH and those with PAH were compared for the impact of endothelin receptor antagonist medication. Following three months of bosentan medication, it was shown that FMD increased in PAH patients but not in CTEPH patients. Nevertheless, FMD did not appear to be correlated with NT-proBNP baseline levels, pulmonary vascular resistance, or the severity of PAH [177].

FMD was lower at baseline in individuals with cardiovascular diseases such heart failure, diabetes, erectile dysfunction, and tobacco use. When PDE-5 inhibitors were administered, FMD increased over time and continued to rise even after the medication was stopped for two weeks [178].

Subsequent studies will need to examine whether the correlation between FMD and all other medicines may also be applied to other recognized treatments for this illness.

## 7. Conclusions

In the upcoming years, the evaluation of endothelial function will become more and more significant, maybe to the point that it will be crucial for both carefully monitoring patients’ conditions and choosing the appropriate course of treatment. It may demonstrate the dual use of endothelial function evaluation as a diagnostic and prognostic tool for patients with PAH. However, it is important to understand that ED examination may not always be immediately applicable or adequate for a conclusive differential diagnosis. Including biomarkers, hemodynamic assessments, and clinical data is still essential for making an accurate diagnosis and selecting the best course of treatment.

## Figures and Tables

**Table 1:** Genes involved in endothelial disfunction in pulmonary arterial hypertension.

Genes associated with the superfamily of TGF-β receptors	BMPR2 is the main gene implicated	ACVRL1
		ENG
		CAV1
		ALK1
		SMAD 1,2,3, 4, 5, 8, 9
		EDN1
		ARRB2
		PTN1
		GDF2
Other genes	Rarer	Transcription factors (TBX4 and SOX17)
		ENO1
		YTHDF2
		EGLN1
		eNOS
		Channel genes (KCNK3, KCNA5, ABCC8, ATP13A3, AQP1, TMEM16)
		ET-1
		EIF2AK4

*BMPR2: Type II receptor of bone morphogenetic protein; ACVRL1: activin receptor type 1; ENG: endoglin; CAV1: Caveolin 1; ALK1: anaplastic lymphoma receptor tyrosine kinase 1; KCNK3: Potassium Two Pore Domain Channel Subfamily K Member 3; EIF2AK4: Eukaryotic Translation Initiation Factor 2 Alpha Kinase 4; SMAD: Mothers Against Decapentaplegic Homologs; TBX4: T-box transcription factor 4; SOX17: SRY-box transcription factor 17; ENO1: Alpha-enolase 1; EGLN1: Egl-9 family hypoxia inducible factor 1; eNOS: Endothelial nitric oxide synthase; KCNA5: Potassium voltage-gated channel subfamily A member 5; ABCC8: ATP-binding cassette subfamily member 8; ATP13A3: ATPase 13A3; AQP1: Aquaporin 1; TMEM16A: Transmembrane Protein 16A; EDN1: Endothelin 1; ARRB2: Beta-arrestin 2; PTPN1: Protein tyrosine phosphatase non-receptor type 1; GDF2: Grow Differentiation Factor 2; YTHDF2: YTH N6-methyladenosine RNA binding protein 2*.

**Table 2:** Aspects of vascular biology.

Cellular changes	Proliferation of pulmonary artery smooth muscle cells (PASMCs) and pulmonary artery endothelial cells (PAECs) contributes to PAH [83].
Typical Lesions in PAH	Plexiform lesions (including onion-skin, plexiform core, and dilation lesions) play a complex role in PAH pathology [85].
Vascular Shunting	Systemic blood vessels (e.g., vasa vasorum, bronchial arteries) may contribute to plexiform vasculopathy [86].
Bronchial-Pulmonary Shunts	Morphometric analysis reveals vascular shunts between bronchial and pulmonary blood vessels in PAH patients [87].
SiMFis Structures	Large fibrovascular singular millimetric fibrovascular lesion (SiMFis) structures connect pulmonary arteries and veins to the systemic circulation [85].
Stem/Progenitor Cells	Stem/progenitor cells may impact PAH progression and repair damaged endothelia [89].
miRNA Dysregulation	Aberrant miRNA expression is linked to apoptosis resistance, inflammation, and vascular proliferation in PAH [92].
miR-204 and miR-21	MiR-204 down-regulation and MiR-21 up-regulation impact PAH pathophysiology [92,93].
Other miRNAs	MiR-22, miR-17/92 cluster, and more contribute to the complex signaling network in PAH.
Endothelin-1 (ET-1)	ET-1 contributes to cellular proliferation and pulmonary artery constriction in PAH [96].
RNA Manipulation	miRNA mimics, antagomirs, and siRNAs aim to precisely regulate gene expression [98,99].
Angiogenesis Modulation	Angiogenic growth factors and inhibitors may restore vascular balance in PAH [98,99].
Stem Cells and Regenerative Medicine	Repairing or replacing damaged pulmonary vascular tissue is now possible [98,99].
Mitochondrial Dysfunction	Mitochondrial metabolism modulators offer potential therapeutic targets [100,101].

PAH: Pulmonary arterial hypertension; SiMFis: singular millimetric fibrovascular lesions; miRNA: microRNA; miR-204: microRNA-204; miR-22: microRNA-22; miR-17-92: microRNA 17-92; miR-21: microRNA-21; ET-1: Endothelin-1; RNA: RiboNucleic Acid; PASMCs: pulmonary artery smooth muscle cells; PAECs: pulmonary artery endothelial cells; siRNAs: small interfering RNAs.

**Table 3:** Methods to evaluate peripheral endothelial function.

NON-INVASIVE	FMD [110]
	L-FMC [116,117]
	ezFMD [136]
	LDF [137]
	RHI [118]
	Doppler Echocardiography [143]
	Magnetic Resonance Imaging [143]
	Positron Emission Tomography [143]
	Pulse Wave Velocity [144]
	Nailfold Videocapillaroscopy [143]
	Near-Infrared Spectroscopy [143]
INVASIVE	Venous Occlusion Plethysmography [38,115,116,117,118,119,120,121,122,123,124]
	EDD using intra-arterial or intravenous infusion [106,107,108,109]
	Angiography [153]

FMD: Flow-mediated dilation; L-FMC: Low-flow-mediated constriction; ezFMD: Enclosed zone FMD; LDF: Laser Doppler flowmetry; RHI: Reactive Hypermia Index; EDD: Endothelium-dependent dilation.
